# Rhythmic Dynamics and Synchronization via Dimensionality Reduction: Application to Human Gait

**DOI:** 10.1371/journal.pcbi.1001033

**Published:** 2010-12-16

**Authors:** Jie Zhang, Kai Zhang, Jianfeng Feng, Michael Small

**Affiliations:** 1 Center for Computational Systems Biology, Fudan University, Shanghai, People's Republic of China; 2 Department of Electronic and Information Engineering, Hong Kong Polytechnic University, Hong Kong, People's Republic of China; 3 Life Science Division, Lawrence Berkeley National Laboratory, Berkeley, California, United States of America; 4 Department of Computer Science and Mathematics, Warwick University, Coventry, United Kingdom; University of Potsdam, Germany

## Abstract

Reliable characterization of locomotor dynamics of human walking is vital to understanding the neuromuscular control of human locomotion and disease diagnosis. However, the inherent oscillation and ubiquity of noise in such non-strictly periodic signals pose great challenges to current methodologies. To this end, we exploit the state-of-the-art technology in pattern recognition and, specifically, dimensionality reduction techniques, and propose to reconstruct and characterize the dynamics accurately on the cycle scale of the signal. This is achieved by deriving a low-dimensional representation of the cycles through global optimization, which effectively preserves the topology of the cycles that are embedded in a high-dimensional Euclidian space. Our approach demonstrates a clear advantage in capturing the intrinsic dynamics and probing the subtle synchronization patterns from uni/bivariate oscillatory signals over traditional methods. Application to human gait data for healthy subjects and diabetics reveals a significant difference in the dynamics of ankle movements and ankle-knee coordination, but not in knee movements. These results indicate that the impaired sensory feedback from the feet due to diabetes does not influence the knee movement in general, and that normal human walking is not critically dependent on the feedback from the peripheral nervous system.

## Author Summary

Complex physiological rhythms arise from a large variety of biological systems that include natural pacemakers as well as feedback mechanisms, from the heartbeat to the rhythmic movement of human walking. Accurately extracting and characterizing the fluctuations underlying the biological rhythms is a fundamental problem which holds the key to understanding the mechanisms that govern the dynamics of biological systems. Usually such signals demonstrate certain oscillatory patterns, with each period displaying irregular fluctuation, or nontrivial dynamics, over time. This renders traditional spectral methods and nonlinear techniques less effective. We propose a novel approach to highlight the intrinsic fluctuations masked by the periodic component and noise through advanced dimension-reduction techniques, and apply it to human gait data from healthy subjects and diabetics. We find that this approach is capable of extracting the intrinsic dynamics and identifying the subtle synchronization pattern between knee and ankle. We find that although the two groups of individuals demonstrate remarkable differences in the dynamics of ankle movement and ankle-knee synchronization, the knee movement of both groups show similar dynamics. These results suggest that sensory feedback from a peripheral nerve system (like the feet) does not play an important role in regulating the motor control of human walking.

## Introduction

Complex physiological rhythms and synchronization processes are ubiquitous in biological systems and are fundamental to life [1]. The human heartbeat [2], [3], walking [4], vocal cords vibration [5], blood pressure and respiration [6], white blood-cell count and tremor in patients [7], [8], epidemic dynamics [9] all demonstrate a stable, possibly nonlinear, oscillatory pattern along with highly irregular fluctuations from period to period. Such signals are variously known as semiperiodic, approximately periodic or pseudoperiodic time series. The fluctuation overlying the oscillatory pattern, or specifically, the cycle-to-cycle variability, arises from the combined effects from the changing environment, the nonlinear nature inherent to biological systems, and noise of various sources. It contains a wealth of information regarding the health or disease status of an individual subject. Usually, little or no *a priori* knowledge or models that govern the underlying system are available. Therefore accurately characterizing and quantifying such biological rhythms through data-driven approaches contributes significantly to our understanding of complex biological control systems [10] and have important applications in disease diagnosis.

Traditionally, rhythmic signals are fruitfully analyzed by linear methods like the Fourier transform and power spectrum analysis. However, physiological signals as outputs of complex biological systems are typically nonlinear and non-stationary, and can not be properly characterized by linear methods. A number of new techniques based on nonlinear dynamical system theory [11], [12] have also been intensively applied, like correlation dimension [13] and Lyapunov exponents [14]. Although the chaotic measures may provide new insights into the nonlinear nature of the system, they are severely hampered by the cyclic trend and noise in the system [15], [16]. Recent attempts include producing pseudoperiodic surrogate data [17], or performing a transformation from time domain to dual complex network domain [18]–[24]. Generally, there still lacks systematic and robust approaches to handle such oscillatory, possibly nonlinear time series. The Fourier analysis decomposes the signal into harmonics that span over the entire time-line, thus information about how each period, or cycle, changes over time has been averaged out. Similarly, nonlinear measures are also based on averaged properties of phase space attractor reconstructed from the data. This lack of discrimination among the individual cycles calls for more advanced signal processing techniques. Inspired by the recent advances in the field of dimensionality reduction [25], [26], we propose a novel and robust approach that can effectively capture the dynamics of cycle-to-cycle variation, which is especially suitable for analyzing approximately periodic, or semiperiodic data like human gait, ECG, respiration, and vowel data.

Human walking is a highly complex, rhythmic process which was found to exhibit long-range correlation and self-similarity, and has attracted sustained interest over the past decades [4], [27]–[33]. The fluctuations overlying the cyclic trend in human walking may reflect valuable information about the neuromuscular processes responsible for normal and pathological locomotor patterns. In particular, the stride interval (SI) (the duration of each gait cycle) has been intensively studied to quantify the physiological or pathological state associated with walking. For example, it has been found that the long range correlation properties are altered with aging and disease [10], [28]. The stride interval reflects the duration of each cycle, and a wealth of information contained in the waveform of the gait cycle is lost. Our approach utilizes the full waveform of each gait cycle, and is therefore expected to extract more information relevant to motor control of walking. We apply our method to the locomotion data collected from two groups of people — healthy subjects and neuropathic patients suffered from diabetes. We aim to find whether the extracted dynamical fluctuation of the knee and ankle movements as well as their synchronization pattern can vary between the healthy and diabetics group. Specifically, we want to find out whether the impaired sensory feedback due to diabetes can lead to different locomotion dynamics of the knee and ankle (and their synchronization pattern) compared with the healthy subjects.

## Methods

### Reconstructing Dynamics Underlying Cyclic Trend: Dimensionality Reduction

The general problem of dimension reduction has a long history. With advances in data collection, dimension reduction has reemerged as a prominent tool to unravel the high dimensional structure emerging in various disciplines. For example, it has been widely applied to gene and protein expression profiling for disease classification and prognostication [25], [26]. Generally, the large number of dimension reduction approaches can be categorized into linear methods, including the principle component analysis, multidimensional scaling [34], and nonlinear methods such as state-of-the-art Isomap [34], laplacian eigenmaps [35] and local linear embedding [34]. Usually biomedical data process nonlinear structures and that nonlinear dimensionality reduction methods might be more appropriate [26]. Here we use *Laplacian Eigenmaps* [35], which are based on *spectral graph theory* and projects the high-dimensional data into a low dimension so that two points nearby on the manifold are kept near to each other. We first illustrate with benchmark data from the chaotic Rössler system described by:
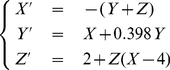
(1)


**Figure 1:**
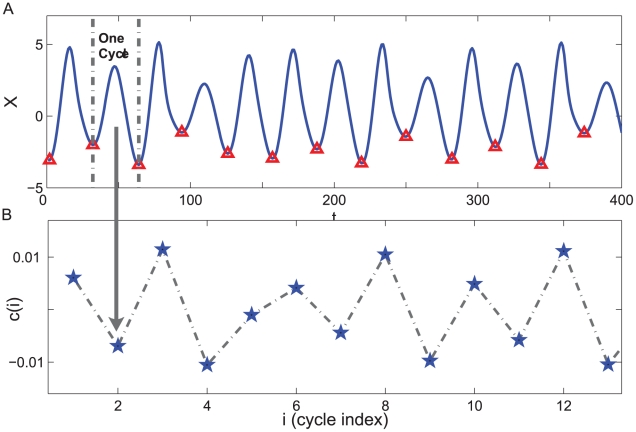
Illustration of transforming a pseudo-periodic time series 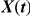 into a new series 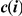 by reducing each cycle in 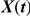 to a point. (A) Time series form 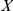-component of the chaotic Rössler system, which demonstrates obvious oscillatory pattern. It can be divided into consecutive cycles at local minimum points (denoted by triangles). (B) A new representation of the oscillatory time series 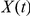 on the cycle scale, with each point in 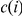 corresponding to a cycle in 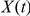.

The time series from the 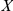-component (see Figure 1A), exhibits a strong periodic component along with irregular fluctuations, therefore it serves as an ideal example of an approximately periodic signal with nontrivial dynamics. Motivated by the fact that such data usually exhibit a highly redundant pattern in the form of repeated cycles, we can partition the time series into individual cycles 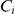 (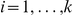, which is the index of the cycle) at the peaks or troughs in the time series, see Figure 1A [36]. Each individual cycle can then be taken as a high dimensional vector 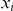 (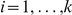), whose dimension equals the number of points in that cycle. Our goal is to map these multiple, high-dimensional cycles to a set of new, low-dimensional (preferably one dimensional) representation, or embedding 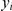
's, such that the proximity relations among 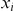
's are maximally preserved in their low-dimensional counterparts 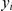
's. In the case that each cycle 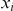 is reduced to 1D (i.e., 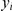 being a scalar), the derived 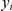 constitute a new time series 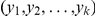, which encodes the dynamics the original time series on the cycle scale.

To achieve this, a weighted matrix 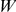 is constructed, with each entry 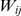 denoting the similarity between cycle 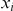 and 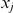, which can be chosen conveniently as the correlation coefficient 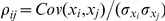 (under the circumstance that 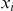 and 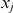 differ in length, we shift the shorter vector along the longer one until 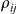 maximizes). Then, the low-dimensional representation 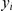
's can be cast as the solution of the following optimization problem, 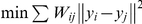, which penalizes those mappings where nearby points 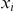
's are relocated far apart in the space of 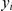
's. In case of univariate 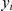
's, the objective can be written as 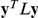, where 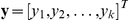, 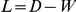 is the *graph Laplacian*, and 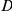 is the diagonal degree matrix such that 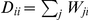.

**Figure 2:**
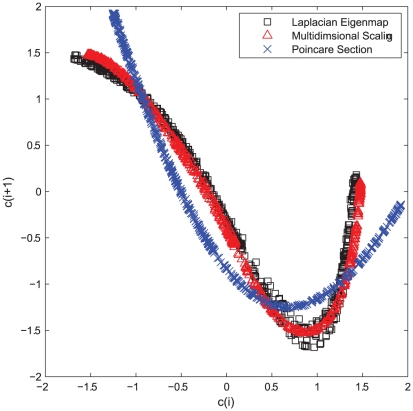
Return maps of 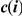 series and Poincaré section points 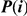. The time series 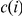 is extracted from the oscillatory data 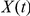 using two dimension reduction techniques, i.e., the Laplacian Eigenmaps and Multidimentional scaling. The Poincaré section points 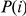 are extracted by collecting the local minimum points of 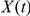.

The above constrained minimization is solved by the generalized eigenvalue problem 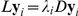, where 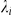
's (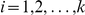) are eigenvalues sorted in an ascending order, and 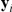
's are the corresponding eigenvectors. The minimum eigenvalue 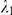 is zero, corresponding to an eigenvector (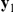) whose entries are all 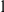. Therefore it is degenerate and the optimal solution is actually provided by 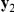, the eigenvector of the second smallest eigenvalue [35]. As is shown in Figure 1B, the eigenvector 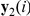 provides a cycle-scale representation of the original time series by reducing each full cycle to a single point. We use a general notation 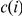 (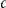 for “cycle”, and 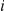 indicates the 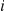
th cycle) for this simplified representation of the original time series. It is worthwhile to note that other dimension reduction schemes can also be adopted. For example, we can compute the Euclidian distance among cycles [36], and multidimensional scaling can be readily applied in this case to reduce the cycles to scalars. The 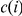 derived from multidimensional scaling and the Laplacian Eigenmap in fact yield quite similar results, see Figure 2.

The applicability of dimension reduction techniques is generally justifiable, considering the low correlation dimension of most real world pseudoperiodic data. For this kind of data, the trajectories of nearby cycles in phase space usually have similar orientations. Such redundancy can be effectively removed through dimension reduction, leaving only the useful degree of freedom. Finally, it is worthwhile to mention that for long time series with large number of cycles, the nyström method can be adopted to solve large scale spectral clustering problem [37], [38].

### The Advantage of Characterizing Dynamics on the Cycle Scale

A popular method in nonlinear time series analysis is to reduce a continues flow to a series of discrete points, called a Poincaré section. The Poincaré section is the intersection of flow data in the state space with a hyperplane transversal to the flow. Thus each cycle in the data is simplified into a single point on the Poincaré section, which preserves many properties of periodic or pseudoperiodic orbits. Now we compare 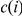 obtained by dimension reduction and the Poincaré section points 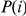 obtained by collecting the local minimum points in the data. As can be seen Figure 2, the return plot (i.e., plot of 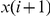 versus its previous values 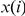) of 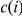 and 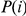 show similar quadratic form. Further calculation of the chaotic measure such as correlation dimension indicates that they have the same dynamical origin.

**Figure 3:**
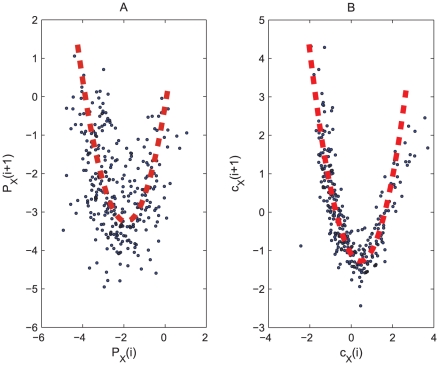
Return plots of Poincaré section points 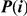 and 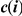. (A) Return plot for 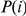 series that is obtained by collecting the local minimums 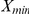. (B) Return plot for 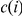 series that is extracted from 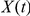 by dimension reduction. The Rössler system here is corrupted by 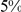 dynamical noise and 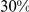 measurement noise. Obviously 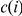 is less influenced by noise than 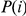.

One problem with the 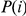 series is that it is highly susceptible to noise that is inevitable in biological data. To see this, we plot the return map for the 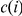 and 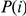 series obtained from the noisy 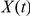 data, see Figure 3. We find that although both return maps display a clear quadratic form intrinsic to the chaotic Rössler system, the return map of 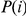 is more vulnerable to noise as the points are more dispersed than that of 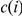. We use the variance 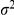 of the least-square fit to the quadratic function (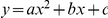) to quantify the influence of noise, with 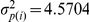, almost 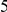 times larger than that of 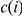 (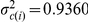). Now we explain why 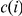 is more robust to noise. First we take the “cycle” as the basic unit rather than a discrete point in the time series. Obviously the latter is more vulnerable to noise. Second, the acquisition of 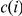 is based on an optimization that preserves the proximity relation among all cycles simultaneously, while 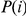 is obtained by treating each cycle independent of one another. Our approach utilizes the richer information of pairwise cycle correlation, therefore it not only excavates the inherent dynamics obscured by the cyclic trend, but also offers an extra robustness to noise due to the global nature of this method.

### Detecting Degree of Synchronization from Bivariate Oscillatory Data

Another interesting phenomena associated with rhythmic process is synchronization between self-sustained oscillators, which plays an important role in understanding coordination or cooperation in biological systems [39], [40]. Several different types of synchronization have been observed, such as complete synchronization [41]–[43] generalized synchronization [44], and phase synchronization [45].

The evaluation of degree of synchronization from the outputs of coupled systems is of particular interest to study the interactions in biological systems. For example, the consistency of mutual nearest neighbors and the peakness of the phase difference distribution are used to characterize the dynamical interdependence [46] and phase synchronization [45], respectively. Here we are interested in the case where two processes are phase synchronized, but the synchronization strength is hard to estimate due to noise and non-phase-coherence, or is too subtle to be differentiated due to the mask of strong phase synchronization. For example, the knee and ankle move perfectly in phase during human walking. Under such circumstances the phase synchronization index will take on high values for both healthy subjects and diabetics and it may not probe the subtle difference in the degree of synchronization masked by the strong phase synchronization and noise.

**Figure 4:**
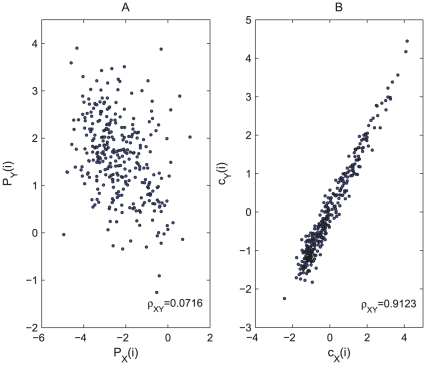
Correlation between 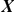 and 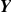 component of noisy Rössler system as is revealed by Poincaré section points 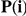 and 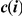. (A) Correlation between 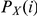 and 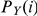. (B) Correlation between 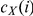 and 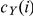. The Rössler system is corrupted by 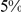 dynamical noise and 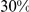 measurement noise.

To solve this problem, we propose to quantify the degree of synchronization between two noisy, phase-synchronized oscillatory processes *on the cycle scale* through the reduced representation 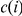 of the original data, thereby minimizing the influence of phase synchronization. We illustrate with the 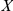 and 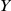 components of the noisy Rössler system, which are perfectly in phase and therefore serve as ideal benchmark data. The two time series are first segmented into cycles according to the local minimums of either 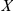 or 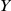 time series, then we apply Laplacian eigenmap to both and obtain reduced representations, i.e., 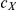 and 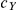 for 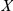 and 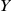 time series. Finally we calculate the linear correlation coefficient 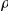 between 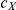 and 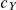 as an indicator of the degree of synchronization between 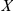 and 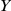 data. We find that the extracted 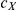 and 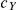 series can successfully reveal the synchronization pattern in presence of noise, which is demonstrated by an increasing trend in the corresponding scatter plot (Figure 4B). The Poincaré section points, however, are less informative of the synchronization degree due to the presence of noise, as 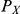 and 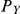 do not demonstrate clear correlation (Figure 4A).

## Results

### Data Description

Now we apply the method proposed in previous section to human gait data collected from two groups: the healthy controls (CO) and neuropathic patients (NP, with significant diabetic neuropathy), each with 10 subjects [4]. The kinematic data were collected from a portable data-logger equipped on the subjects during continuous overground walking for 10 minutes (sampled at 66.7 Hz). Three electrogoniometers were placed on the approximate joint centers of the hip, knee, and ankle joints of the right leg to measure their sagittal plane movements. Here we consider the signals measured from knee and ankle joints movement.

### Characterizing Human Locomotion Dynamics

**Figure 5:**
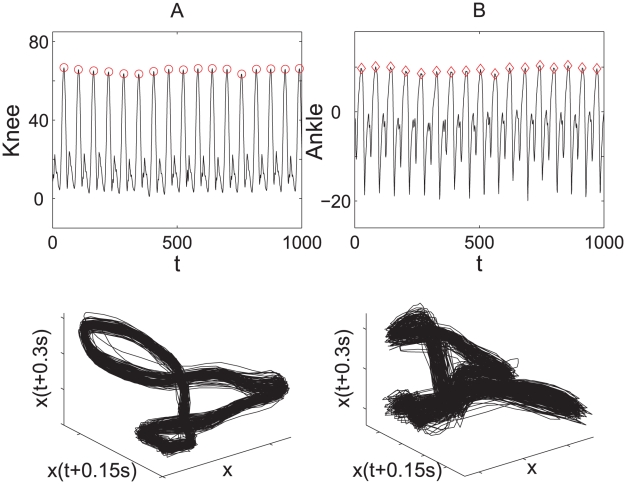
Time series (upper panel) and the corresponding phase space reconstructions (lower panel) of knee and ankle locomotion from a healthy subject. (A) Knee locomotion data. (B) Ankle locomotion data. The time series are typically non-phase-coherent, demonstrating multi-oscillation within each cycle. This is also evident from the multi-center rotations of the attractor in phase space (lower panel). The two time series are divided into consecutive cycles by their respective local maximum points.

Human locomotion is a highly complex, rhythmic process that involves control from subcortical locomotor brain regions and feedback from various peripheral sensors. Typically, the human gait time series (see Figure 5 for knee and ankle movement) exhibit a stable frequency while irregular stride-to-stride fluctuation. For biological signals with a strong periodic component, vital information regarding the pathology and pathophysiology of the subject is hidden in the *cycle-to-cycle variation*. Accurately extracting this fluctuation and characterizing its dynamics are expected to provide important insights into the underlying neuromuscular control of walking and yield greater diagnostic information. For example, the stride interval (SI), defined as the time duration of each gait cycle, has been widely used to study the human gait [27], [28], [31]–[33]. It was shown that SI series displays long-range correlation intrinsic to the healthy locomotor system.

**Figure 6:**
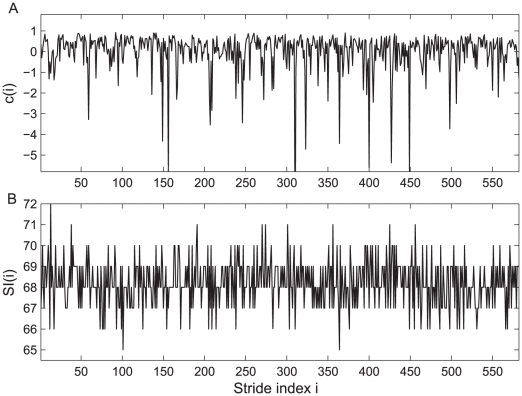
Time series of the extracted 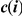 and the stride interval (SI) from ankle movement data of a healthy subject. (A) 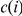 time series, which demonstrates significant fluctuation that is not observed in original ankle movement data. (B) Stride interval series extracted from the ankle movement data, which, due to digitization in data collection, loses much information about the original dynamics.

The SI series contains the information of the duration of each gait cycle. Another source of information consists in the waveforms of the gait cycles, which is not reflected in SI series [29]. As can be seen in Figure 6, the stride interval from the gait data seems to contain insufficient information to reflect the intrinsic dynamics due to digitization. Therefore it is natural to expect that the 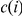 series, which is obtained by comparing the waveforms of each cycle (therefore preserves the full dynamical pattern within each cycle) will contain more information relevant to the locomotion dynamics. Meanwhile, 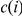 successfully removes the periodic trend that may obscure the underlying dynamics, and has the same advantage as SI series. In the following we will demonstrate that 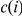 obtained by Laplacian Eigenmap can reveal the dynamical fluctuation underlying the cyclic trend more effectively than the SI series, so that we can distinguish clearly between the healthy and pathological groups and make inference about the neuromuscular control, especially on the role of sensory feedback from the feet in regulating dynamics of human walking.

**Figure 7:**
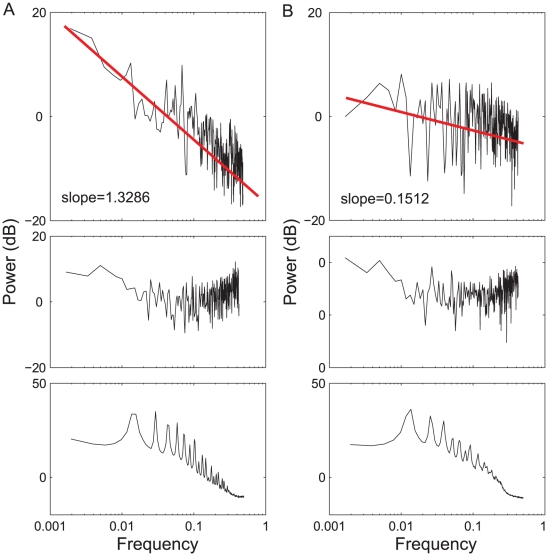
Power spectrum density (PSD) for a typical healthy subject (the left panel) and a diabetes patient (the right panel). (A) A healthy subject. (B) A diabetes patient. The top, middle and bottom rows are PSDs for the extracted 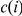, stride interval series, and the original ankle data, respectively. It is obvious that the PSDs for the stride interval series and the original data show no significant difference between the healthy subject and the diabetic patient. The sampling rate of 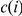 can be taken as the mean stride interval, and the log-log PSDs are then fitted with a linear function using least-square regression.

**Figure 8:**
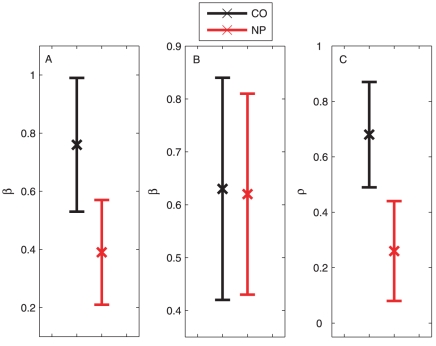
Mean and standard deviation of the derived statistics for control (CO) and neuropathic (NP) groups. (A) The slope 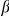 of the least-square-fit to the power spectrum density for the ankle movements. (B) The slope 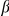 of the least-square fit to the power spectrum density for the knee movements. (C) The correlation coefficient 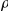 between 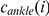 and 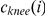.

First we check the ankle movement (see Figure 5B), and use the Laplacian eigenmap to extract the fluctuation 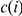 on cycle scale for the two groups. To quantitatively characterize the time evolution of 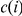, we furthermore compute its power spectrum density (PSD), see the top row in Figure 7. We find that most CO subjects demonstrate broad band spectrums (i.e., 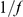 noise) that scale as 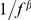, with 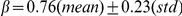 (see Figure 8A), indicating the presence of long range correlation (i.e., the strides separated by a large time span are still statistically correlated). In comparison, the power spectrum of the diabetic patients are mostly flat resembling white noise processes (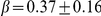), which means that the strides at different times are mostly uncorrelated. The values of 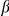 in the two groups are statistically different (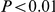). This difference, however, has not been found with either the stride interval (SI) series (see the middle row in Figure 7) or the raw data (see the bottom row in Figure 7).

The absence of long range correlation in the ankle kinematics of the NP group suggests the alteration of the locomotor pattern in the lower limbs of neuropathy patients. This is due to the loss of peripheral sensation in the lower limbs, which arises from the gradual dying back of nerves from the fingers and toes typical of diabetes. Despite the deterioration in peripheral nervous system, the *knee* movement of the NP group is *still* found to demonstrate a stable long range correlation indistinguishable from the CO group (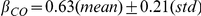, 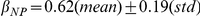, which are statitcally identical with 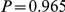), see Figure 8B. These results suggest that the impaired peripheral feedback caused by the dying nerves in the feet does not influence the upper-limb dynamics, which leds us to another fundamental problem in human walking, i.e., what is the role of sensory feedback in adjusting the global locomotor dynamics? To understand this, we examine further the degree of synchronization between the knee and ankle movement using dimension reduction.

### Assessing Synchronization between Knee and Ankle Movement

Human walking involves the coordination of two major joints, i.e., the knee and the ankle, whose movements during continuous walking are obviously in phase due to the physical connection between them. However, we find that correlation between knee and ankle movement for the two groups can hardly be distinguished by the phase index of the signal due to the presence of strong phase synchronization. Also, noise tends to destroy the local structure in phase space and thus hampers the dynamical dependence measures [47]. To circumvent these difficulties, we propose to compare the dynamics of the two time series by using their Laplacian eigenmap 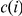
's. Note that each time series can be segmented by either its own local maximums, or those of its partner series (shown in Figure 5). Therefore we will segment each time series twice and compute the averaged correlation coefficients 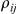
's between 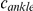 and 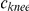 for these two segmentation schemes.

**Figure 9:**
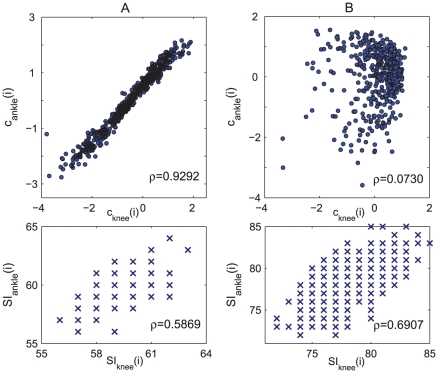
Synchronization pattern between knee and ankle locomotion revealed by 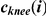 and 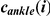
(upper panel) and stride interval series 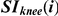 and 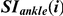 (lower panel). (A) A healthy subject. (B) A diabetes patient. As can be seen in the lower panel, the stride interval series 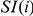 cannot distinguish the healthy from the diabetics, as the scatter plots between 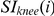 and 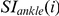 show similar increasing trends for both subjects.

Figure 9A shows the typical synchronization pattern between ankle and knee movement for healthy subjects. The scatter plot between 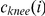 and 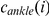 demonstrates a significant increasing trend, indicating that the knee and ankle movements are highly synchronized. The correlation coefficient 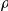 between 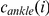 and 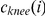 for the healthy group takes on a high value: 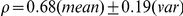, see Figure 8C. For diabetics, however, there is little correlation between 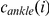 and 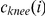, as is manifested in randomly distributed points in Figure 9B. The correlation coefficient in this case is also low: 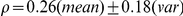, and the values of 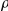 is statistically different for the two groups (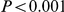). All the results of 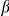 and 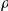 values for the two groups of individuals are summarized in Supplementary Table S1. Again, the discrimination between CO and NP groups cannot be achieved by SI series, which always exhibits a strong correlation between the two joints (Figure 9, lower panel), corresponding to high degree of phase synchronization. Finally, we point out that a more comprehensive description of synchronization can be achieved by examining more Laplacian eigenvectors. In the current case the single eigenvector 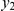 already encodes the primary variability and is thus sufficient for the discriminative task.

The lack of significant synchronization between ankle and knee movements observed in diabetic patients suggests the “incoordination” between the ankle and knee movements. This may arise from the gradual deterioration of the nerves in foot and toes of the diabetics, which is unable to produce sufficient neural feedback for the lower limb to be coordinated with the upper limb. In section “Characterizing Human Locomotion Dynamics” we have found that both the ankle and knee movements for healthy subjects demonstrate long range correlation, while for patients, only the knee movement show long range correlation. This finding is consistent with the result obtained here, i.e., the ankle and knee movement are more synchronized for healthy people than for the diabetics. Finally it should be noted that our study is limited by the relatively small sample size (each group has 10 subjects). Therefore significance tests are performed to verify the differences observed between the two groups of individuals. The current conclusion will be further validated on a larger data base available in the future.

## Discussion

### Central Nerve Control over Peripheral Nerve System

A fundamental question concerning human walking is the origin of the long range correlation (or 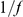 noise) found in human gait data, the mechanism of which are not exactly clear [28]. Generally, the locomotor system incorporates inputs from both the central nervous system such as the motor cortex and basal ganglia, and peripheral inputs and sensory feedbacks. Both these two kinds of inputs are suggested to be possible reasons for the presence of the long range correlation in normal human walking [4].

In section “Characterizing Human Locomotion Dynamics”, we found that although the locomotion dynamics of the ankle shows significant difference between the normal persons and the patients in terms of long range correlation, their knee movements demonstrate similar scaling properties. These results support the belief that the impaired peripheral feedback from the sensors in the feet of diabetics influences only the lower limb locomotion while not that of the knees. We therefore conclude that human walking is not critically dependent on the feedback from peripheral feedback of the lower-limb, and that the central nervous system is playing a major role in regulating locomotor dynamics. In fact it has been found that pathology in central nervous system, such as Huntington's disease, can result in a loss of long range correlation in the gait dynamics [28]. For diabetics, although the peripheral sensory feedback is weakened, their central nervous system is not damaged and still plays an important role in adjusting the locomotion dynamics. This is why their knee locomotion still demonstrate 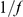 dynamics. Finally, it was pointed out that diabetics may still retain proximal somatosensory inputs, and visual or vestibular feedback information [33]. Further study need to be done to clarify the role of these factors in regulating the dynamics of human walking.

### Possible Application to Other Areas

Our approach may be of great relevance, and is expected to provide more accurate and robust characterization and diagnostics to the complex oscillatory data observed in general biological and engineering fields. Reconstructing the dynamics on the cycle scale also brings new vitality to a number of other approaches which are otherwise not suitable for analyzing rhythmic data directly, such as detrended fluctuation analysis [48], recurrence plot [20]–[22], [49], entropy measures [50]–[52], surrogate data method [17], causality analysis[53]–[56], and so on. The inherent periodicity in time series can cover up the intrinsic dynamical fluctuation, thus extracting the dynamics on a cycle scale is crucial for subsequent analysis. For example, human vowel data and sunspot number variation are typical oscillatory time series in biomedical and astrophysical fields. We can easily extract the corresponding 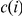 series from such signals and feed them into DFA algorithm. By this we are able to tell if the original data has long range correlation on the cycle scale more reliably than other approaches.

Another striking example of physiological rhythms and their interaction is the complex, human cardiovascular system (CVS) [57], [58]. For example, the human heart is driving the blood circulation, in which one heartbeat corresponds to one cycle of blood pressure variation. Meanwhile, the heart rate is modulated by respiration through the so called Respiratory Sinus Arrhythmia (RSA). Analyzing the interaction among these biological rhythms from simultaneously measured ECG, blood pressure, and respiration force is crucial for understanding the cardiorespiratory control and disease diagnostics. Due to noise and non-phase-coherence of ECG, blood pressure and respiration signal, traditional measures may not be able to capture the subtle changes in the degree of synchronization, which could possibly be probed more accurately by our approach.

Finally, it is worthwhile to note that our approach can also be applied to time-course microarray data [59], as well as Functional Magnetic Resonance Imaging (FMRI) time series [61]–[63]. For example, genes related to cell-cycle control are always expressed periodically and subject to fluctuation, thus reliably characterizing the correlation or synchronization pattern among such genes using microarray data is expected to provide more insights into the corresponding regulatory network responsible for cell-cycle transcription and regulation. Similarly, FMRI experiments, which are unusually designed with periodic stimuli, always lead to periodic BOLD responses demonstrating large fluctuations [64]–[66]. Our approach is therefore expected to evaluate more reliably the functional connectivity among different regions in the brain for a deeper understanding of cerebral function.

## Supporting Information

Table S1 β and ρ values for Control (CO) and Neuropathic (NP) groups.
(0.03 MB PDF)
