# Supplementary material for: Rhythmic Dynamics and Synchronization via Dimensionality Reduction: Application to Human Gait
Source: PLoS Comput Biol. 2010 Dec 16;6(12):e1001033. doi: 10.1371/journal.pcbi.1001033 (PMC3002994; doi:10.1371/journal.pcbi.1001033)
Supplement: Table S1 — β and ρ values for Control (CO) and Neuropathic (NP) groups. (0.03 MB PDF) [file pcbi.1001033.s001.pdf]

Table 1. β and ρ values for Control (CO) and Neuropathic (NP) groups

	CO group			NP group	
β for c_{ankle}	β for c_{knee}	ρ	β for c_{ankle}	β for c_{knee}	ρ
0.9187	0.9369	0.8044	0.1512	0.6056	0.1683
1.3286	0.9720	0.9292	0.3247	0.7074	0.1681
0.6232	0.5710	0.8348	0.5959	0.8876	0.6432
0.7473	0.7867	0.6836	0.4163	0.5106	0.2611
0.7589	0.4013	0.3300	0.6609	0.8478	0.3650
0.7190	0.4559	0.4028	0.3166	0.6950	0.0730
0.4838	0.6204	0.7423	0.3680	0.2490	0.1680
0.5893	0.3996	0.6271	0.2738	0.4910	0.3810
0.6730	0.5645	0.6882	0.5345	0.5901	0.0563
0.7153	0.6319	0.7523	0.2966	0.6653	0.3434
